# Early abduction treatment versus observation in Barlow-positive and mildly unstable hips

**DOI:** 10.1186/s12887-025-05940-x

**Published:** 2025-08-04

**Authors:** Vilma Lankinen, Mika Helminen, Karim Bakti, Jarmo Välipakka, Hannele Laivuori, Anna Hyvärinen

**Affiliations:** 1 Department of Pediatric Surgery, Turku University Hospital, Savitehtaankatu 5, Turku, 20520 Finland; 2 Faculty of Medicine and Health Technology, Tampere University, Tampere, Finland; 3 Faculty of Social Sciences, Health Sciences, Tampere University, Tampere, Finland; 4 Tays Research Services, Tampere University Hospital, Tampere, Finland; 5 Pihlajalinna, Tampere, Finland; 6 Faculty of Medicine and Health Technology, Center for Child, Adolescent, and Maternal Health Research, Tampere University, Tampere, Finland; 7 Department of Obstetrics and Gynecology, Tampere University Hospital, Tampere, Finland; 8 Institute for Molecular Medicine Finland (FIMM), Helsinki Institute of Life Science, University of Helsinki, Helsinki, Finland; 9 Medical and Clinical Genetics, University of Helsinki and Helsinki University Hospital, Helsinki, Finland; 10 Department of Surgery, Mehiläinen Länsi-Pohja Oy, Kemi, Finland; 11 Department of Pediatric Surgery, Oulu University Hospital, Oulu, Finland; 12 Clinical Medicine Research Unit, Medical Research Center, University of Oulu, Oulu, Finland; 13 Department of Surgery, Tampere University Hospital, Tampere, Finland

**Keywords:** DDH, Barlow positive, Watchful waiting strategy, Spontaneous recovery

## Abstract

**Background:**

In the treatment of DDH, stable but dysplastic hips are safe to observe, and these children do not usually need abduction treatment. It has been reported, that also clinically unstable hips have good spontaneous recovery potential, but only a few studies have investigated the observation strategy in clinically mildly unstable (Barlow positive) hips. A conclusion on the safe treatment strategy for these children has not been made.

**Materials and methods:**

All early diagnosed mildly unstable (Ortolani negative) hips treated in Tampere University Hospital in 1998–2018 were found, and data was retrospectively collected from the medical records. A total of 510 children were found. There were 222 children with Barlow-positive hips of which 45% were first observed, and 288 children with reported clinically mild hip instability but no reported Barlow-positivity of which 90% were first observed. All the analyses were done separately for these two groups of children.

**Results:**

Girls were more likely to need abduction treatment after observation in Barlow-positive and mildly unstable groups. There were no differences in the six-month alpha angle or treatment failure rates between early-treated and first-observed children in either of the study groups. Duration of the treatment was not increased in observed children in either of the study groups.

**Conclusion:**

Observation for about a month in clinically mildly unstable hips with or without Barlow positive signs seems safe regarding the recovery of alpha angles, treatment duration and treatment failures. More research is needed for longer observation times.

## Introduction

Developmental dysplasia of the hips (DDH) includes a spectrum of conditions from stable hip dysplasia to severe instability and total dislocation of the hip joint [1]. It is recommended, that dislocated hips should be treated early after birth, as late diagnosis and treatment might predispose to abduction treatment failure, long casting periods, and operative treatment [1, 2]. It has been documented, that stable but dysplastic hips, diagnosed with ultrasound, can be safely observed, as these hips usually recover without treatment [3]. With these children, waiting the onset of the treatment for approximately one month has been found safe, regarding the abduction treatment duration as well as treatment failures and residual hip dysplasia. These children seem to recover well even if they would need abduction treatment later [3, 4]. Literature has also described that hips with mild clinical instability have good recovery potential and 40–88% of these hips have been documented to recover without treatment [5, 6]. The majority of the recent studies around the watchful waiting strategy have been mainly focused on stable but dysplastic hips [3, 4, 7], and there are only few studies that have studied watchful waiting strategy in clinically unstable hips [5, 8]. We wanted to investigate this treatment strategy further and see if observation of clinically mildly unstable hips is safe regarding the correction of alpha angles, treatment failures, and abduction treatment duration.

## Materials and methods

All the children with a diagnosis of developmental dysplasia of the hips (ICD10 Q65.0-Q65.9) treated in Tampere University Hospital in the years 1998–2018 were found, and data was collected retrospectively from the medical records. Altogether 948 children with a DDH diagnosis were found. As we wanted to investigate the safety of the observation strategy in early diagnosed clinically mildly unstable hips, we excluded all the completely dislocated hips (Ortolani-positive sign) (*n* = 389) from the analysis. Teratological dislocations (*n* = 3) were also excluded. Children diagnosed at seven weeks or after were excluded (*n* = 46). Only the children with early diagnosis of DDH (within the first six weeks of life) by a pediatric surgeon/resident or a pediatric orthopedic surgeon/resident were included. All in all, 510 children with clinically mild instability were found.

In Tampere University Hospital we have selective ultrasound screening for DDH. Ultrasound is done approximately at the age of one month. In the years 1998–2000, a Frejka pillow was mainly used for abduction and after the year 2000, Pavlik Harness became the main course of treatment.

Hips were either treated with early abduction or observed for approximately 1 month. The decision of the firstly selected treatment was made by the clinician. After one month of age, clinical status together with ultrasound findings guided the treatment. An ultrasound was performed every 4 weeks until the hips were found normal (alpha angle 60 degrees or over). In addition, all the children underwent the last ultrasound checkup at approximately 6 months of age.

Early treatment was defined as abduction treatment starting within the first two weeks of life. Delayed treatment was defined as all the abduction treatments starting after two weeks. Abduction treatment was initiated by a pediatric surgeon/surgery resident or a pediatric orthopedic surgeon. The Pavlik harness was worn 24 h/day and was not removed by the families during the treatment. Harness adjustments were done every two weeks of treatment by a pediatric surgeon/surgery resident or a pediatric orthopedic surgeon. The follow-up protocol was the same regarding the Frejka pillow, which was worn 23–24 h/day, and only taken off during diaper changes.

Positive family history was defined as one or more first-degree relatives (parents or siblings) with a diagnosis of DDH.

In the first clinical status, hips were reported by the clinicians to be either Barlow-positive, mildly unstable in provocation without Barlow positivity or mildly unstable with no mention of Barlow positivity. We decided to do all the analyses separately to the children with clear Barlow positive sign and to children with only reported mild instability. We included the children with no mention of Barlow positivity into the group of children with mildly unstable hips. Mild instability was defined as any minor instability/laxity mentioned by the clinicians while doing the Ortolani and Barlow tests, but no positive test signs, characterized by dislocation without provocation (Ortolani positive) or only with provocation (Barlow positive).

Abduction treatment failure was defined as any other treatment (casting or operation) after abduction.

The abduction treatment duration was reported as days.

All the analyses were done by using SPSS 23. The associations between different treatments (observation, early abduction, delayed abduction) and the correction of alpha angles, and treatment failure were evaluated in cross-tabulations with chi-square statistics. The deviation of normality was checked on the treatment duration. As it was not normally distributed, the Mann-Whitney test was used for binominal variables and Kruskal-Wallis for multinominal variables for statistical analyses in the bivariate models. All the analyses were controlled in multivariable models. The binary logistic regression model was used with categorized variables and the linear regression model with continuous variables. Due to the skewness of the treatment duration distribution, the variable’s logarithm was used in the regression model. Raw residuals were considered symmetrical, marking the reliability of the selected method.

## Results

There were 275 (53.9%) children who had mildly unstable hips, 222 (43.5%) children with Barlow-positive hips and 13 (2.5%) children with no mention on Barlow positivity (these children were included in to mildly unstable group in the analyses). The majority (69.2%) of children were girls. See all the patient demographics in Table 1. The majority of children (92.5%) were diagnosed within the first two weeks of age (range 0–48 days).

**Table 1:** Patient demographics

Patient characteristics (*n* = 510)		*n* (%)
First clinical status of the hip	Barlow positive Mildly unstable Missing information on Barlow positivity	222 (43.5%) 275 (53.9%) 13 (2.5%)
Treatment	Early abduction treatment (within 2 weeks) Delayed abduction treatment Only observation (clinical/radiological follow-ups) Operation and/or casting	146 (28.6%) 153 (30.0%) 211 (41.4%) 5 (1%)
Sex	Male Female	157 (30.8%) 353 (69.2%)
Presentation	Breech presentation Cephalic presentation Information missing	142 (27.8%) 366 (71.8%) 2 (0.4%)
Family history	Positive Negative Information missing	73 (14.3%) 252 (49.4%) 185 (36.3%)
6 months alpha angle	under 43 43–49 50–59 60 or over Information missing	0 (0%) 1 (0.2%) 9 (1.8%) 424 (83.1%) 76 (14.9%)
Abduction duration	1–30 days 31–60 days 61 days or over No abduction treatment Information missing	165 (32.4%) 117 (22.9%) 16 (3.1%) 211 (41.4%) 1 (0.2%)

**Fig. 1:**
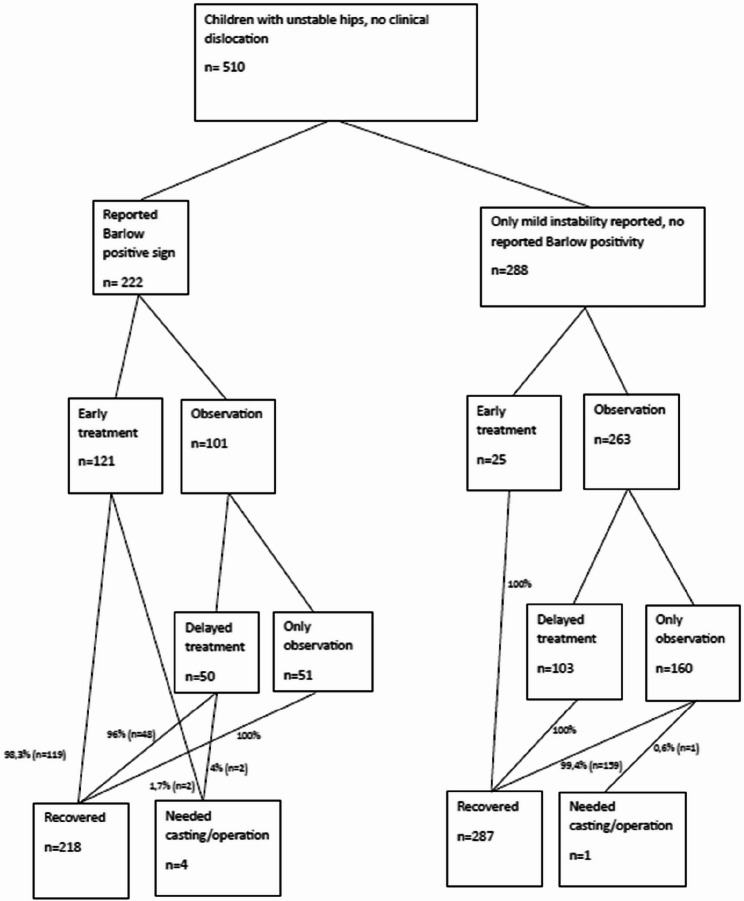
Flow chart of the patients

In the early treatment group, the mean age for treatment start was 4 days (range 1–14 days), and in 96% of cases the treatment was started within the first week of life. The mean time of diagnosis was 3.4 days (range 0–14 days) and median was 3 days. In the delayed treatment group, the mean observation period was 33 days (range 15–64 days) and 88% of the children were observed for four weeks or more before the treatment started. The mean time of diagnosis was 6.7 days (range 0–47 days) and median was 3 days. In Barlow-positive children, 121 (54.5%) were early treated and 101 (45.5%) were observed initially. In mildly unstable hips 25 (8.7%) were treated early and 263 (91.3%) were observed. Of the observed children 49.5% (*n* = 50) needed treatment in the Barlow positive group and 39.2% (*n* = 103) in the mild instability group. Only 5 children (0.99%) needed casting and/or operation. See patient flow in the Fig. 1.

The treatment groups (early abduction and observation) were similar regarding sex, breech presentation and family history of DDH in Barlow-positive and in mildly unstable children. Girls were more likely to need abduction after observation in Barlow-positive (*p* = 0.023) and in mildly unstable groups (*p* = 0.025). These results are presented in Table 2, respectively.

**Table 2:** Risk factors and treatment of Barlow-positive and mildly unstable hips. All of the variables were assessed together in the multivariable models

	Barlow positive					Mild instability				
Risk factor	Treatment			*p*-value	*p*-value in multivariable model	Treatment			*p*-value	*p*-value in multivariable model
	Early abduction	Delayed abduction	Only observation			Early abduction	Delayed abduction	Only observation		
Sex				0.023	0.006				0.025	0.007
F	84 (54.5%)	41 (26.6%)	29 (18.8%)			19 (9.5%)	80 (40.2%)	100 (50.3%)		
M	37 (54.4%)	9 (13.2%)	22 (32.4%)			6 (6.7%)	23 (25.8%)	60 (67.4%)		
Breech presentation				0.436	0.848				0.575	0.229
Yes	42 (60.9%)	13 (18.8%)	14 (20.3%)			7 (9.6%)	29 (39.7%)	37 (50.7%)		
No	79 (51.6%)	37 (24.2%)	37 (24.2%)			17 (8.0%)	73 (34.3%)	123 (57.7%)		
Family history				0.847	0.865				0.629	0.594
Yes	15 (46.9%)	9 (28.1%)	8 (20%)			4 (9.8%)	15 (36.6%)	22 (53.7%)		
No	57 (53.8%)	24 (22.6%)	25 (23.6%)			10 (6.8%)	57 (39.0%)	79 (54.1%)		
Information missing	49 (58.3%)	17 (20.2%)	18 (21.4%)			11 (10.9%)	31 (30.7%)	59 (58.4%)		

At six months of age, no statistically significant differentiation in alpha angles was found between early treated children and the children who were selected for observation in Barlow positive (*p* = 0.469), nor in mildly unstable (0.599) groups. Risk factors did not affect the alpha angle at six months of age. See the results in Table 3

**Table 3:** Observation strategys and risk factors affect on the recovery of alpha angles at 6 months of age. All of the variables were assessed together in the multivariable model

	Barlow positive				Mild instability			
Variable	Alpha angle at 6 months of age		*p*-value	*p*-value in multivariable model	Alpha angle at 6 months of age		*p*-value	*p*-value in multivariable model
	< 60	60 or over			< 60	60 or over		
Treatment			0.469	0.128			0.599	0.755
Early abduction	3 (2.8%)	106 (97.2%)			0 (0%)	20 (100%)		
Observation strategy	4 (4.7%)	81 (95.3%)			3 (1.4%)	217 (98.6%)		
Risk factor								
Sex			0.868	0.845			0.235	0.999
F	5 (3.8%)	128 (96.2%)			3 (1.8%)	161 (98.2%)		
M	2 (3.3%)	59 (96.7%)			0 (0%)	76 (100%)		
Breech presentation			0.529	0.543			0.306	0.999
Yes	3 (4.8%)	59 (95.2%)			0 (0%)	61 (100%)		
No	4 (3%)	128 (97%)			3 (1.7%)	174 (98.3%)		
Family history			0.496	0.526			0.253	1.0
Yes	1 (3.7%)	26 (96.3%)			0 (0%)	35 (100%)		
No	2 (2.1%)	93 (97.9%)			3 (2.4%)	123 (97.6%)		
Information missing	4 (5.6%)	68 (94.4%)			0 (0%)	79 (100%)		

There was no differentiation in the duration of treatment between early treated and children with delayed treatment in the Barlow positive group (*p* = 0.064) nor in the mildly unstable group (*p* = 0.522). In the Barlow positive group of children girls needed longer treatments than boys (*p* = 0.011) and breech-born children recovered faster than cephalic-born children (0.016). In the mildly unstable group, risk factors did not affect the treatment duration. The results are presented in Table 4.

**Table 4:** Observation strategys and risk factors effect on the duration of abduction treatment. All of the variables were assessed together in the multivariable model

	Barlow positive				Mild instability			
Variable (n)	Treatment duration (days)		*p*-value	*p*-value in multivariable model	Treatment duration (days)		*p*-value	*p*-value in multivariable model
	Mean	Median			Mean	Median		
Treatment			0.064	0.215			0.522	0.481
Early abduction (	37.5	33			33.8	28		
Delayed abduction	36.2	29			35.6	29		
Risk factor								
Sex			0.011	< 0.001			0.653	0.423
F	38.7	32			35.6	29		
M	32.9	29			34.0	28		
Breech presentation			0.016	0.005			0.289	0.194
Yes	32.1	30			33	28		
No	39.5	33			36.1	29		
Family history			0.437	0.072			0.805	0.333
Yes	41.9	32.5			37.6	28.5		
No	35.0	30			33.8	28		
Information missing	38.1	31			36.6	29		

In Barlow positive children, there were no differences in treatment failure between early treated and observed children (*p* = 0.314) as only four children needed casting/operation, 2 in the early treatment group and 2 in the observation group. All of these children were diagnosed within the first 5 days of life. Only one of the children (observation, no treatment) needed casting and/or operation in mildly unstable group, in this case the diagnosis was set at 22 days of age. These results can be seen in Table 5.

**Table 5:** Observation strategys and risk factors effect on the failure of the abduction treatment. All of the variables were assessed together in the multivariable model

	Barlow positive				Mild instability		
Variable	Treatment failure		*p*-value	*p*-value in multivariable model	Treatment failure		*p*-value
	No	Yes			No	Yes	
Treatment			0.314	0.436			0.669
Early abduction	119 (98.3%)	2 (1.7%)			25 (100%)	0 (0%)	
Delayed abduction	48 (96%)	2 (4.0%)			103 (100%)	0 (0%)	
Observation	51 (100%)	0 (0%)			159 (99.4%)	1 (0.6%)	
Risk factor							
Sex			0.805	0.822			0.134
F	151 (98.1%)	3(1.9%)			199 (100%)	0 (0%)	
M	57 (98.5%)	1 (1.5%)			88 (98.9%)	1 (1.1%)	
Breech presentation			0.175	0.316			0.087
Yes	69 (100%)	0 (0%)			72 (98.6%)	1 (1.4%)	
No	149 (97.4%)	4 (2.6%)			213 (100%)	0 (0%)	
Family history			0.058	0.118			0.395
Yes	30 (93.8%)	2 (6.3%)			41 (100%)	0 (0%)	
No	106 (100%)	0 (0%)			146 (100%)	0 (0%)	
Information missing	82 (97.6%)	2 (2.4%)			100 (99%)	1 (1%)	

All of the analyses were controlled in multivariable models, and it did not change the direction of the significations. The failure of the treatment in the mildly unstable group could not be controlled by a multivariable model as there was only one child failing the treatment in this group. The *p*-values from the multivariable models can be seen in the Tables 2, 3, 4 and 5.

## Discussion

According to our results, it seems that the observation strategy of clinically mildly unstable hips (with or without Barlow positive sign) is safe regarding treatment duration, correction of alpha angles and failure of the abduction treatment. Our findings are in line with earlier findings regarding the subject [5, 8]. Barlow found in 1962, that the majority of clinically unstable hips stabilize without treatment [6]. Still, it is vastly recommended that clinically unstable hips should be treated early, because the late diagnosis and treatment in DDH have been associated with treatment failure. With clinically mildly unstable hips, a conclusion about the treatment initiation time has not been made [1, 9–11]. 

Rosendahl et al. found, that stable dysplastic hips recover well without treatment, but their study sample was mainly sonographically abnormal hips as only 20% of their children were clinically mildly unstable [4]. Laborie et al. had the same conclusion in their prospective study, but they also had mainly sonographically abnormal, stable but dysplastic hips [7]. Cook et al. observed a group of Barlow-positive children, and 40% of them did not need treatment, and the observation was safe as all of their children recovered [5]. Our results add to these previous studies. Our results are similar to Cook et al., however, our study sample was larger and we still made the same conclusion. Regarding the correction of alpha angles, we add to the study by Cook et al. In our sample five children needed casting and/or operative treatment, but the initiation time of treatment did not seem to affect the risk.

In our data, it seemed that girls were more likely to need abduction treatment after the observation period, and Barlow-positive girls also had longer treatments than boys. These findings are similar to our earlier findings regarding DDH risk factors [12–14]. Girls are thought to have an increased risk of DDH due to hormonal factors affecting the ligament laxity. It seems logical, that due to this same reason girls might have more severe instability than boys and thus they might need more time and/or more robust treatments to recover. In the observation strategy, it is important to inform families, that girls might have a bigger risk than boys to need abduction treatment even after the observation period, however, observation is still safe regarding the recovery of alpha angles and treatment failure.

Breech-born infants recovered faster than cephalic-born infants. This finding is also in line with our previous studies [12–14]. As breech presentation is purely a mechanical risk factor, it seems logical that in these children, hips have great potential to recover fast as the risk factor is removed at birth. Our results indicate, that breech-born infants do not automatically need abduction treatment if the initial instability is minor, and it is safe to observe these children in the beginning.

In our data, four Barlow-positive children needed operation/casting, and 2 of them (50%) had positive family history. Earlier, we have found, that positive family history might increase the risk of longer abduction treatments and abduction treatment failure [12, 13]. In this study there was no significant associations between positive family history and treatment duration nor treatment failure. This might be due to the smaller number of patients in this study. There were also a number of patients missing the information regarding the family history, which also could affect the results. In the analysis, there still was a trend towards signifigance regarding positive family history and treatment duration as well as treatment failure. We think that positive family history might be an important factor in DDH treatment and these children should be treated early with abduction, if the clinical instability is present, to minimize the risk of treatment failure.

We did not find any associations between the risk factors and treatment duration nor failure in the mildly unstable group of children without Barlow positive sign. We think that these children had very minimal instability to begin with, and the children in Barlow positive group had generally more significant instability. It seems that the common risk factors of DDH affect mainly the initial severity of the instability, which then has an affect on the recovery. In very minor cases the risk factors do not seem to matter, as almost all of these children recovered quickly, regardless of the treatment initiation time or risk factors of DDH. Only one child needed operating in this group (mildly unstable hips without Barlow positivity), and we think that in this case, the first clinical diagnosis into the group of mild instability might have been incorrect. All in all, the early clinical diagnosis seemed effective, as there were only 5 children in the whole data failing the treatment in the study period of 20 years.

Our research has some limitations. As the data was retrospectively collected, the exact reasons for clinicians’ decisions for choosing their treatment strategies cannot be known. However, in our data there were no statistically significant differences regarding the main risk factors between the children treated early and those observed in the beginning. In addition, due to the retrospective study design, the treatment initiation times were not standard, and we decided to define early start at the age of two weeks or under. This could lead to the similarity of these two treatment groups regarding the treatment initiation times. However, in our data majority of the early treated children (96%) had the treatment start within the first week of life, and on the other hand majority (88%) of the children in delayed abduction had at least four weeks of observation time before treatment initiation. This clearly separates these treatment groups. Another limitation is the definition of mild instability. The description of the clinical instability of the hips is not always clear, and in some cases Barlow positivity was not reported in our data. As the severity of DDH is a spectrum, the exact definition of the clinical instability might sometimes be difficult. Clinicians may have variations in the description of mild instability. For this reason, we decided to do the analyses separately on the children who were reported to be Barlow-positive and children who were only reported to have mildly unstable hips, and we found the same results for both of the groups. Regarding the effects of the risk factors, it seemed that the Barlow positive group had more unstable hips to begin with, compared to the mildly unstable group in our data. Clinical diagnosis of DDH is always somewhat ambiguous, however, this is common practice worldwide [15], and ultrasound screening seems to add overtreatment and thus, is not generally recommended [15, 16]. 

Sometimes the diagnosis of DDH may be delayed. It has been reported that treatment starting after seven weeks of age may increase the risk of treatment failure [17]. To avoid the confounding factor of late diagnosis, we defined early diagnosis as under 7 weeks of age; however, the majority of children (92.5%) in our study were diagnosed within the first two weeks of age. The time of diagnosis did not affect the treatment failure, as four of the children who failed were diagnosed within the first 5 days of life, and one child who failed was diagnosed at 22 days of age. We think that as long as the hips are not completely dislocated (characterized as Ortolani positive sign), the spontaneous recovery potential is good, and the waiting period of four to six weeks seems safe. The current evidence suggests that stable but dysplastic hips do not necessarily need abduction treatment to recover. We think that it also might be safe to follow the hips with mild instability for an even longer period of time. However, confirmation of safety for the longer waiting periods can not be made according to this study, and more research is needed on the matter.

## Conclusion

Early diagnosed Barlow positive and/or mild hip instability is safe to be observed for a month regarding the treatment duration, treatment failure and recovery of alpha angles.

## Data Availability

The datasets generated and/or analyzed during the current study are not publicly available due to patient privacy and confidentiality but are available from the corresponding author on reasonable request.

## Abbreviation

DDH: Developmental dysplasia of the hip
